# Late Complications and Adverse Events in Adult Deformity Surgery: A Narrative Review of Event Types, Prevalence, and Information Gaps

**DOI:** 10.1177/21925682251342556

**Published:** 2025-07-09

**Authors:** Sigurd H. Berven, Justin S. Smith, John T. Street, Eric Klineberg, Yong Qiu, Aboubacar Wague, Stephen J. Lewis

**Affiliations:** 1 Department of Neurosurgery and Orthopaedic Surgery, 8785 University of California San Francisco, Spinal Disorders Service, San Francisco, CA, USA; 2 Department of Neurosurgery, 2358 University of Virginia, Charlottesville, VA, USA; 3 Department of Orthopedic Surgery, 8166 University of British Columbia, Vancouver, BC, Canada; 4 Departments of Orthopedic Surgery, University of Texas Health Houston, Houston TX, USA; 5 Department of Orthopaedics, The Affiliated Drum Tower Hospital, Nanjing University Medical School, Nanjing, China; 6 Department of Orthopedic Surgery, 8785 University of California San Francisco, San Francisco, CA, USA; 7 Department of Surgery and Spine Program, Toronto Western Hospital, University of Toronto, 7989 University Health Network, Toronto, ON, Canada

**Keywords:** adult spinal deformity, surgical complications, PJK, infection, pseudoarthrosis

## Abstract

**Study Design:**

Literature review.

**Objectives:**

The purpose of this paper is to provide a narrative review of late complications in adult deformity surgery, including infection, pseudarthrosis and junctional pathology after deformity correction. This review aims to highlight limitations of current management and identify potential areas of improvement and further study.

**Methods:**

We identified common challenges of late complications in adult spinal deformity surgery and performed a directed literature review to summarize the current management and issues encountered in these adverse events. Through consensus, we highlighted the knowledge gaps in the current literature and suggested areas of interest for further study to improve understanding and management of these conditions.

**Results:**

A summary is provided with detailed review of late complications that include infection, pseudarthrosis, junctional pathology, and late decompensation after deformity correction. Important consideration to choosing the appropriate upper and lower instrumented levels, obtaining desired coronal and sagittal alignment, preserving the adjacent ligamentous and muscular structures, obtaining solid arthrodesis, recognizing and optimizing patients for surgery, appropriate pre and post-operative protocols, and appropriately defining normal and pathological parameters.

**Conclusions:**

Recognizing appropriate predictor and outcome variables are important for identifying factors, modifiable and fixed, that may be important to make a clinically important difference in outcomes in the surgical treatment of adult spinal deformity. Future studies to reduce late complications are important to improve value and outcome in adult spinal deformity surgery.

## Introduction

Adult spinal deformity (ASD) is an important priority for health systems internationally based upon the expanding aging population and the impact of spinal deformity on health status of affected patients.^1-4^ The management of adult spinal deformity is characterized by significant variability, encompassing options of non-operative and operative care.^5,6^ Internationally, trends in spinal surgery demonstrate a significant increase in both the frequency and the complexity of surgery for adults with spinal deformity.^7-12^ The appropriate use of surgery for elderly patients with spinal deformity is an important health system priority.^13^ The appropriateness of multilevel surgery for deformity in adults has not been well defined.^14^ An appropriate surgery, as defined by the UCLA/RAND appropriateness method, is a surgery where the expected benefit of the procedure outweighs the anticipated risks.^15^ Multiple studies have demonstrated improvement of health status outcomes after adult spinal deformity surgery.^16-19^ Complication rates of multilevel fusion surgery in adults have been consistently high, and the ratio of risk to benefit has been variable and debatable.^20-22^ Surgical complications in deformity patients have been reported with significant variability in the literature, with variability enforced by limitations of retrospective data collection, voluntary reporting of data, and limited follow-up.^23-31^ Accurate information regarding complications in adult patients undergoing multilevel fusion surgery for spinal deformity is valuable for empowering informed choice by patients and physicians. The purpose of this paper is to provide a narrative review of late complications in adult deformity surgery, including delayed surgical site infection, pseudarthrosis, and junctional pathology after deformity correction and to determine gaps in our current knowledge that would be amenable to future study.

## Materials and Methods

A consensus meeting was undertaken to review the most important late complications related to the surgical treatment of ASD. The authors considered a broad range of complications and chose to focus the review on complications that were significant in adult deformity surgery and about which there is not a clear consensus regarding avoidance and treatment. Following review, the authors chose to review late surgical site infection, pseudarthrosis and implant failure, proximal junctional failure and distal junctional failure. The narrative review technique included identification of primary studies on each topic with the goal of summarizing each late complication with information on diagnostic criteria, incidence, management, and strategies for avoidance. An important goal of the narrative review was to identify areas of controversy and gaps in the existing literature in order to identify areas of interest for future study. The narrative review is not as comprehensive as a systematic review, and the selection of primary studies was made in order to summarize the topic rather than to analyze published data to provide new conclusions.

## Results

### Late Complication: Infection

Surgical site infection in spine surgery may be an early or late complication. Late infections are infections that occur more than 3 months after the index procedure and are the focus of this review. The diagnosis, prevention and management of Delayed Surgical Site Infection (DSSI) following Adult Spinal Deformity Surgery (ASDS) is hindered by a lack of standardized nomenclature and definition, and a number of fundamental knowledge gaps of this diverse, complex and growing condition. ASDS is of itself a heterogenous term with myriad surgical indications including adolescent idiopathic scoliosis in adulthood with no prior surgery, iatrogenic flatback, and revision surgery for pseudarthrosis, to name but a few. As a result, the procedures and techniques of ASDS are also highly variable, including minimally invasive lateral procedures with percutaneous posterior screw fixation (MIS), open posterior pedicle screw-based constructs with multiple Ponté type osteotomies and major posterior based osteotomies including pedicle subtraction osteotomy and vertebral column resection (OPEN) or a combination of techniques (HYBRID). While the heterogeneity in indication and procedure renders identification of and mitigation against risk factors for DSSI challenging, equally challenging are the fundamental estimations of prevalence and etiology.

### Incidence and Etiology

The incidence of late surgical site infections has been reported with significant variation. The complication is significant regarding cost and impact on the patient, and therefore is included as a topic of this narrative review. According to a systematic review by Zanirato in 2018 (96 publications including 12 168 patients), late deep surgical site infection (presenting more than 3 months after the index procedure) had an incidence of 0.18% in OPEN procedures, was unreported in HYBRID, and was 0.31% in MIS procedures.^32^ Based on the definition proposed in this systematic review, deep surgical site infection could be considered an extremely uncommon postoperative complication (incidence <0.5%). This study is likely representative of the general ASDS population, as the mean age was 54.08 ± 12.94 years, mean follow-up was 3.34 ± 1.43 years, mean EBL was 1828.11 ± 956.59 mL, mean number of fused levels was 7.82 ± 2.66, and the mean operative time was 370.99 ± 161.67 min, though the finding of a modestly higher incidence of infection in MIS cases may be surprising. However, only 13 of the 96 studies were prospective, and definitions of perioperative and late as well as major and minor varied considerably across studies.^33^

Smith et al assessed the rates of complications associated with 291 patients ASDS with minimum 2-year follow-up based on a multicenter study design that incorporated standardized data-collection forms, on-site study coordinators, and regular auditing of data.^34^ The mean age was 56.2 years (SD 15.2 years), and the distribution of patients by age group was as follows: 18-44 years, 19.2%; 45-64 years, 48.1%; and 65-86 years, 32.6%. The perioperative rate of infection was 10.7% within 6 weeks, without delayed infections identified in the subset of patients with 2 year follow-up.

Lewkonia et al retrospectively reviewed a prospectively collected database and reported an incidence of DSSI (delayed defined as > 90 days) of 0.12% from a cohort of 5770 surgical procedures (including revision degenerative and deformity cases).^35^ Of the 7 patients with a DSSI, only a single patient lacked an obvious risk factor for infection, such as prolonged wound drainage, a chronic remote infection, or an initial infection in a remote site during the early post-operative period. These authors also surveyed an international group of spine surgeons to determine their routine practice in either recommending or not recommending the use of prophylactic antibiotics for patients undergoing invasive medical procedures such as colonoscopy or dental work, following a prior spinal fusion. Although the survey of opinions revealed some differences in expert recommendations in certain specific circumstances, approximately 2/3 of experts would not recommend prophylactic antibiotics for patients with an uncomplicated previous lumbar instrumentation.^35^

Increasingly, index ASDS includes revision surgery for conditions such as iatrogenic flat back, pseudarthrosis and other forms of mechanical failure. While the incidence of DSSI for ASDS specifically for revision has not yet been examined, the incidence of early SSI in revision spinal fusion has been estimated at 2-9%, compared to 1-4% for primary fusion surgeries with Odds Ratios (OR’s) ranging from 1.5 to 2.5. These OR’s likely represent at least a minimum if not an underestimate of the risk of DSSI in ASDS for revision.

### Diagnosis

The diagnosis of DSSI poses a number of unique challenges. For the purposes of this discussion, we have limited our scope to Deep SSI (Category II) as defined by the CDC guidelines.^36^ Based on these guidelines, Deep SSI involves deep soft tissues within the incision, occurs within 1 year if implant is in place and infection appears to be directly related to surgical procedure, and must fulfill one of the following additional criteria: (a) purulent drainage from incision but not from the organ/space of the site; (b) dehiscence or deliberate opening by the surgeon from the deep incision when the patient has at least one of the following signs or symptoms of clinical infection (fever greater than 100.4°F, localized pain or edema, unless culture is negative); (c) abscess or other evidence of infection involving the deep incision is found during examination of incision, reoperation, or pathologic or radiologic exam; (d) diagnosis of a deep incisional SSI by a surgeon or attending physician. Our collective clinical experiences and the limited available literature suggest that DSSI following ASDS primarily manifests as (c) or (d) above. While it is commonly accepted that ‘delayed’ implies more than 3 months from the date of the index surgical procedure, there is no definitive consensus and thus the literature is heterogeneous even in the fundamental area of defining DSSI.

In practicality, DSSI typically presents in the form of back pain, often without constitutional symptoms and may also have wound drainage. The hallmark findings of acute infection are can be absent, and a high index of suspicion must be present in all forms of mechanical failure, particularly if otherwise unexpected and occurring between 6 weeks and 6 months following the index procedure. Microbiological diagnosis prior to any revision surgery is critical, to ensure optimal pre-operative planning. Diagnosis of post instrumentation infections may benefit from CT- or fluoroscopic-guided deep aspiration. However, this has a low diagnostic yield of around 40% when compared to cultures from intraoperative infected tissue, often because of previous antibiotics. The diagnostic yield of these cultures can be optimized by the use of a large bore needle, histopathological examination as an adjunct to cultures and a delay in antibiotic therapy until after the biopsy. At the time of revision surgery, multiple tissue samples should be routinely sent for pathological and microbiological examination. This should include prolonged culture (>5 days) to identify pathogens that are slowly growing. The use of additional staining for fungi and mycobacteria is advised, especially with previous negative culture results.

In their article in 2019, Agkun et al demonstrated that serum C reactive protein showed low sensitivity and specificity for the diagnosis of delayed postoperative spinal implant infection even after applying cut offs optimized by using receiver operating curve analysis, because of the high incidence of low virulent pathogens.^37^ Those authors found a significant difference in serum CRP levels between septic and aseptic cohorts (19.3 vs 4.8 mg/l, *P* < 0.001). However, 43% of patients from the ‘infected’ group had a normal (<5 mg/l) serum CRP level prior to revision surgery. According to the ROC curve, a serum CRP threshold of 4.05 mg/l only had a sensitivity of 64% and a specificity of 68%. Despite this, inflammatory markers are still important diagnostic tools, and comparing pre-operative with post-operative levels, and following trends over time, should be done when suspecting SSIs following spine surgery.

Radiological examination may demonstrate loose instrumentation, failure of fusion or loss of deformity correction, however there is no gold standard imaging modality to make the diagnosis of DSSI. While magnetic resonance imaging remains the diagnostic modality of choice when suspecting acute SSI following spine surgery, it has limited utility in delayed infection. Radionuclide imaging has shown higher sensitivity and earlier detection of acute SSI compared to CT scanning and X-ray imaging. For instance, Gallium67 scanning can show focal increased uptake in areas suggestive of infection with high sensitivity and specificity of 89 and 85%, respectively. Moreover, it is estimated that the interval for the appearance of diagnostic radiological signs of SSI is shortened with Gallium67 compared to Technetium99 scanning, rendering Gallium67 the preferred agent for early detection of SSI radiological changes. While 18F-fluorodeoxyglucose-positron emission tomography is not widely used, it may be useful in challenging cases. None of these modalities has been specifically examined in the setting of DSSI in ASDS.

In many cases of DSSI, given the benign clinical presentation and paucity of definitive diagnostic tests, the only radiological findings may be loose instrumentation or pseudarthrosis with clinical symptoms of pain. Simultaneous DSSI should be suspected when there is more than expected local bone loss or endplate resorption or if the mode or degree of mechanical failure appears ‘excessive’.

### Strategies for Avoidance

The first step to developing strategies for avoidance of DSSI in ASDS is to identify modifiable risk factors for its development. Again, this has not been reported specifically for DSSI in ASDS. However, there is probably much that can be inferred from studies on early SSI in deformity surgery and delayed SSI in spinal surgery in general. Examining these studies, we can extrapolate the factors common to all spine surgeries and the factors uniquely related to adult deformity surgery. A weighted categorization of these modifiable risk factors would be key to the development of practice changes and quality improvement initiatives to reduce the incidence of DSSI in ASDS.

In their 2018 study, Yao et al performed a literature review of risk factors for surgical site infection.^38^ Patient associated factors included diabetes mellitus, body mass index more than 35 kilograms per meter squared, subcutaneous fat thickness, multiple medical comorbidities, current smoking and malnutrition defined by serum albumin <3.5 mg/dl. Subcutaneous fat thickness more than 50 mm is associated with a four fold increase in infection rates. Surgery-associated factors included pre-operative radiation, post-operative blood transfusion, combined anterior and posterior surgical approaches, and high surgical invasiveness. There was mixed evidence for each of the following: duration of surgery, composition of the surgical team, intra-operative blood loss, dural tear and post operative urinary tract infection.

Chahoud et al also added to this list of risk factors the following: steroid use, alcohol abuse, extremes of age, extent of fusion performed, revision intervention (compared to primary intervention), traditional open approach (compared to minimally invasive approach), omission of drain usage post spine surgery, operative duration greater than 3 hours, stainless steel instrumentation alloy (compared to titanium use).^39^ The authors emphasized the importance of surgical site infections regarding morbidity and economic costs, and provide an algorithm for the diagnosis of surgical site infections that includes clinical evaluation, laboratory studies (inflammatory markers and serum amyloid A), radiographic studies and tissue aspiration.

Apart from the basic principles of debridement and antibiotic therapy, there are a number of unique challenges in the management of DSSI in ASDS. In 2020 Agarwal et al performed a literature review on the necessity for implant removal in the management of surgical site infection after spine surgery.^40^ They reviewed a total of 49 articles. The most common organisms detected were MRSA and *Staphylococcus epidermis*. Long-term antibiotic administration and continuous irrigation and debridement were common treatment suggestions among authors, however the key measure undertaken or implied by most authors to avoid risk of recurrence of infection was removal or replacement of the implants for late onset surgical site infection. Obviously, removal of the instrumentation is usually not possible in situations of major adult deformity, at least not until solid fusion has occurred perhaps 18 to 24 months postoperatively. In the setting of a long fusion, the risk of post implant removal fracture and deformity progression, while not well defined, must be considered. However, implant removal and a short 5-7 day course of no-implants may be necessary to clear these chronic infections. Maruo and Berven demonstrated high rates of recurrent infection after primary irrigation and debridement in cases with late infection (>3 months after index surgery), long instrumented fusions, polymicrobial infections, and *Propionibacterium acnes* infections. Removal of implants and direct or staged re-implantation may be a useful strategy in cases with high risk of treatment failure.^1^ Protection of the fusion mass and correction with bracing during this holiday is crucial. Re-implantation can then be performed with low density implants to decrease the biological burden. Newer implants including titanium and those with special microbiological surface treatment may provide an opportunity to retain the instrumentation and successfully eradicate the infection. Biomaterials may be modified to confer antimicrobial properties through processes of surface coating or surface modifications.^2^ In some situations, choosing to live with a low grade or indolent infection will often be preferable to consideration of removal of the hardware.

Many knowledge gaps exist in our knowledge of DSSI in ASDS, primarily because of the very low incidence and lack of consensus on definition, diagnosis and management. Much of what we know or assume is borrowed from both the literature on adolescent deformity surgery, and that of spinal surgery in general. Single-center or short-term multicenter studies will likely not answer the most key questions:

1. What is the true incidence of DSSI in ASDS, and when, and how does it manifest in a clinically relevant way?
2. What are the most reliable diagnostic tools to determine the occurrence, severity and etiology of DSSI?
3. What is the difference, with respect to etiology and risk stratification, between DSSI in the setting of a solid fusion and DSSI that mandates instrumentation removal and revision reconstruction surgery?
4. What would be the components of a QI bundle to mitigate against the occurrence of DSSI?
5. What effect does DSSI have on health-related quality of life (HRQOL) of patients following ASDS?

### Late Complication: Pseudarthrosis and Implant Failure

Spinal deformity surgical correction requires the utilization of implants to correct the malalignment. Achieving the proper alignment has been shown to improve outcomes and decrease complications. Once the implants create the correction, they must maintain that alignment until a solid arthrodesis can be achieved. Failure to achieve a fusion, may result in pain or late implant failure with rod or screw breakage, and potential need for revision surgery.

Soroceanu et al reviewed the adult spinal deformity database from the International Spine Study Group (ISSG) and reported that 32% (79 of 246) of patients developed an implant or radiographic-identified complication, which led to a 53% reoperation rate.^41^ Rod breakage (n = 16, 20.2%) and PJK (n = 24, 30.4%) were the most common complications. Smith and colleagues reported 24% (71/291) of patients required reoperation at 2 years, primarily due to implant or radiographic related complications, which included implant failure and pseudarthrosis.^42^ Several ISSG prospective series of surgically treated deformity patients report a 9.0% rod fracture rate at 1 year and a 13.7% rate at 2 years.^42,43^ The highest rate of rod fracture was seen in patients undergoing pedicle subtraction osteotomy (PSO), with 22% of patients exhibiting rod fracture vs 4.7% in those without PSO.^43^

When compared to patients without radiographic or implant-related complications, these patients had greater BMI, more comorbidities, and were more likely to have had previous operations.^42^ Patients with radiographic-identified complications tended to have greater preoperative pelvic tilt (PT), greater mismatch between pelvic incidence and lumbar lordosis (PI-LL), and greater sagittal malalignment.^41^ Significant risk factors included older age, greater BMI, history of previous spine surgery, PSO, greater baseline sagittal spinopelvic malalignment (SVA, PT, and PI-LL mismatch), and greater magnitude of sagittal spinopelvic malalignment correction with surgery (SVA and PI-LL mismatch).^42^

There is significant literature dedicated to minimizing the risk of rod fracture and pseudarthrosis. There are data regarding the use of supplemental rod constructs across 3-column osteotomy sites that has found reduced rates of rod failure and pseudarthrosis.^44^ Additionally, achieving the appropriate sagittal correction has both improved clinical outcomes and decreased rates of complications, including rod fracture.^45^ This has led to the development of optimal alignment targets to help minimize implant failure.^45-52^ The final surgical intervention is the controversial use of rhBMP-2 to improve spinal fusion. There have been several cost utility studies demonstrating its cost effectiveness, and safety in adult spinal deformity.^53-57^ Numerous studies have reviewed patient and surgical risk factors associated with the development of PJK and pseudarthrosis.^58-61^ These risk factors have been used in a predictive model with 91% accuracy for detecting pseudarthrosis that can be used for both patient selection and counseling.^62^

Despite this vast body of literature, there remain significant gaps in our knowledge for the prevention of pseudarthrosis and implant failure. Clearly, this is multifactorial problem, and understanding the most important reason for failure in each case is difficult. Additionally, the definition of a nonunion is often debatable, as it can be difficult to identify via radiographs alone and must often be assumed when associated with implant failure, or confirmed with computed tomography (CT). Finally, the ability to use rhBMP-2 and additional rods may not always be feasible due to the significant up-front costs, even if they can help prevent complications later.

The knowledge gap for strategies to limit pseudarthrosis in adult spinal deformity surgery involves both comparative information regarding biologics and bone graft materials, as well as an evidence-based approach to appropriate construct stiffness. Multicenter evaluation of risk factors for implant failure and pseudarthrosis in an international cohort would be useful for study generalizability as risk factors may be different due to cultural and societal differences. A predictive model that takes into account the patient specific factors and construct factors would be useful to guide an evidence-based approach to care. Additionally, a risk- and cost-benefit analysis on the use of biologics, iliac crest autograft or another adjunctive measure to achieve fusion would also be important, especially for patients and surgeons in less resourced countries.

### Late Complication: Proximal Junctional Pathology

One of the greatest unsolved challenges in ASDS is the development of PJK.^6,63^ Based on the relatively liberal Glattes definition (proximal junctional angle >10°),^64^ the overall incidence of PJK has been reported to range from 17% to 62%.^65^ PJK is often asymptomatic and without apparent consequence, but in more severe forms it can be a major source of pain, neurological compromise, and disability, and in some cases may require extensive revision surgery.^66^

Although PJK is most commonly recognized as an early event following surgery for ASD, it can also present several months or years following surgery and has been suggested to follow a bimodal pattern (<3 months and >2 years).^32,34,67^ Segreto and colleagues recently reported the incidence of acute (within 6 weeks of surgery), progressive (increase in degree of PJK from 6 weeks to 1 year), and delayed (1-, 2-, and 3-year) PJK over an 8-year period based on 1005 ASD patients with a mean age of 59 years.^68^ The overall incidence of acute PJK was 48.0%, and the incidence of progressive PJK was 35%. PJK continued to develop at 1-, 2-, and 3-year follow-up, with incidences of 9.3%, 4.3%, and 1.8%, respectively. Yagi and colleagues reported similar findings based on a retrospective review of 76 ASD patients with a mean age of 49 years and a mean follow-up of 7.3 years (range 5-14 years).^69^ The overall incidence of PJK in their series was 22.4%, with 76% of the cases of PJK identified within 3 months of surgery. Notably, 53% of the total degree of PJK developed within 3 months of surgery, with the remaining PJK occurring through continuous progression up to the time of final follow-up.

Multiple risk factors for PJK have been suggested, including older age, low bone mineral density, over or under correction of lumbar lordosis, over-correction (flattening) of thoracic kyphosis, fusion to the sacrum/ilium, and disruption of supportive structures at the upper-most instrumented vertebra.^6,64,68-70^ It remains unclear whether there are distinct risk factors for acute vs delayed PJK. Based on the studies of Yagi and colleagues and Segreto and colleagues, it appears that at least a subset of late PJK occurs through gradual progression of the proximal junctional angle, suggesting that early occurrence of mild PJK may be a risk factor for delayed development of more significant PJK. It is possible that delayed PJK may also develop as a result of risk factors that are partially mitigated by more favorable factors. For example, a patient who has over-correction of lumbar lordosis may be relatively protected from PJK in the acute period by favorable bone mineral density and a strong soft tissue envelope, but cumulative stress and degeneration at the junction over time may ultimately lead to delayed PJK.

Although it remains an area in need of further investigation, it is likely that strategies and techniques aimed to reduce the occurrence of early PJK are also likely to be effective in reducing the development of delayed PJK.^6,71^ Achieving appropriate overall global alignment, without over or under correction of lumbar lordosis and without excessive flattening of thoracic kyphosis, appears to be a critical factor in preventing PJK.^72,73^ Based on 679 ASD patients with fusions extending to the pelvis, Lafage and colleagues reported that patients who were over-corrected based on age-adjusted alignment goals were significantly more likely to develop PJK.^73^ The orientation of the upper-most instrumented segment also appears to be an important factor in development of PJK.^74,75^ Lafage and colleagues reported that ASD patients who developed PJK had a more posterior construct inclination, arguing that proper rod contouring (ie, mild kyphosis) at the proximal end may help to reduce the risk of PJK.^74^ Line and colleagues performed a propensity score matched analysis of 625 surgically treated ASD patients based on development of PJK. They found that use of surgical implants (cement, hook, tether) alone at the UIV to prevent proximal junctional failure was less effective than combining implants with avoidance of sagittal overcorrection.^76^ Yagi and colleagues reported on the use of teriparatide as a protective treatment following ASD surgery in women with osteoporosis and reported a significant reduction in the occurrence of fracture at the UIV.^77,78^

The major knowledge gaps and need for future research on proximal junctional kyphosis as a late complication include:

1) Prospective study to correlate proximal junctional alignment changes with symptoms and function following ASD surgery in order to provide a more clinically meaningful definition of PJK.
2) Precise identification of risk factors for PJK.
3) Prospective investigation of techniques to reduce PJK occurrence with multiple surgeons and centers that specialize in ASD surgery, all applying the state of the art in alignment goals and bone density optimization, along with use of prophylactic measures (cement, hooks, tethers) in isolation or in combination.
4) Collection of granular clinical and operative data, along with long-term follow-up in an effort to identify risk factors and account for long term outcomes.

### Late Complication: Distal Junctional Pathology

#### Prevalence and Etiology

While the literature is replete in the investigation of proximal junctional kyphosis/failure (PJK/PJF), relatively few studies have focused on distal junctional kyphosis/failure (DJK/DJF) in the past decades. Lowe^79^ firstly described DJK, ≥10° of kyphosis between the lower instrumented vertebra (LIV) and LIV-1, as a complication following corrective surgery usually for adolescent idiopathic scoliosis or cervical deformity.

**Figure 1.**
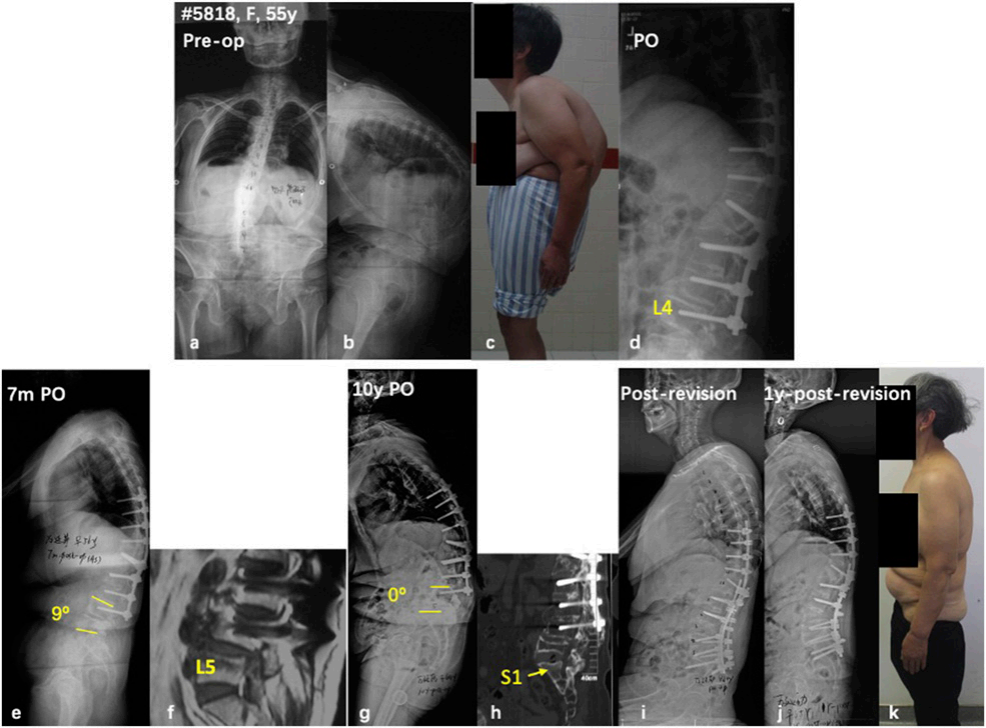
A 55-year-old female with diffusive idiopathic skeletal hyperostosis (a-c). She underwent pedicle subtraction osteotomy at L1 and instrumentation from T8 to L4 (d). At 7-month follow-up, loss of lordosis and retrolisthesis was observed at L4/5 (e, f). Positive sagittal imbalance, L4/5 disc breakdown, and L5 compression fracture was observed at 10 years follow-up (g, h). With pedicle subtraction osteotomy at L4, and pelvic fixation via S2 alar-iliac screws, the sagittal balance was well restored (i). Some loss of correction was observed at 1-year follow-up (j), but the patient remains happy clinically (k).

**Figure 2.**
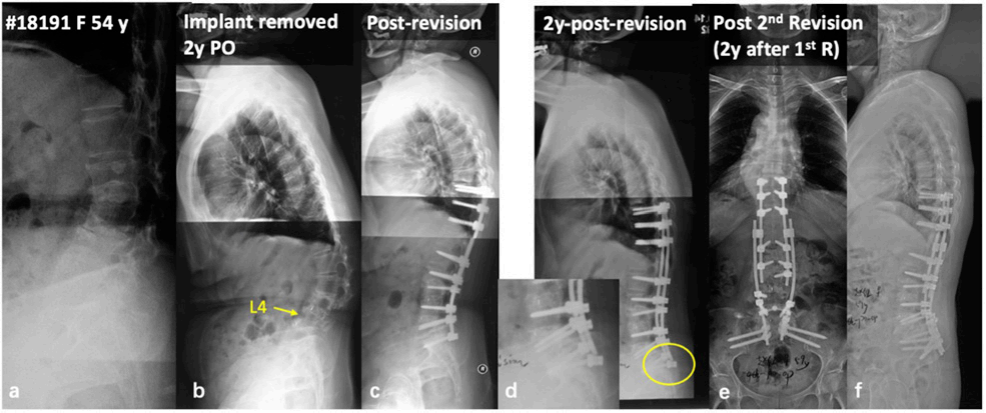
(A) 54-year-old female with degenerative lumbar kyphosis (A); In a local hospital, she underwent TLIF at L3/4 and L4/5, and then her posterior instrumentation was removed 1 year after the initial surgery for unknown reason (B); 1 year later, she underwent revision surgery in another hospital due to progressive lumbar kyphosis (C); 2 years after this revision, she presented with distal instrumentation failure and fixed positive sagittal imbalance (D); During the second revision surgery, we strengthened the distal fixation via bilateral dual S2 alar-iliac screws, and the sagittal alignment was restored and well maintained (E, F).

According to the previous studies, DJK occurs in 4.1%∼15.0% of patients after correction surgery for spinal deformities of various etiologies.^79-85^ In adult spinal deformity (ASD), the reported prevalence ranged from 4.1% to 13.3%.^82-85^ Although the occurrence of DJK is lower than PJK, ASD patients who develop DJK are more likely to receive revision surgeries due to progressive global sagittal malalignment, focal pain and neurological symptoms.^86,87^ In 2013, Arlet^88^ stratified DJK/DJF into six models: (1) Progressive loss of lumbar lordosis, intervertebral height, and degeneration of discs, which could be associated with flat back syndrome and spinal stenosis; (2) Acute disc wedging below LIV^80^; (3) LIV fracture, often involving the inferior endplate of LIV; (4) Osteoporotic fracture below the long rigid instrumentation, often in the lower lumbar spine (Figure 1), and may also involve the sacrum; (5) Instrumentation failure at LIV (Figure 2)^83^; (6) Spinal stenosis and or segmental instability under the instrumented mass, retrolisthesis and anterolisthesis could be observed. Considering the increasing trend of the aging population and the high prevalence of ASD, an increasing number of DJK patients could be expected in the future.^89,90^ To date, however, few peer-reviewed studies have systematically investigated DJK in the spectrum of ASD.

Improper selection of LIV has been associated with the development of DJK. Previous studies have demonstrated that long fusion to L5 was associated with loss of lordosis in the distal junctional segment.^83,91^ Kwon^83^ retrospectively analyzed the radiographic and clinical data of 13 ASD patients, 11 (84.6%) of whom had mechanical failures at L5 or the L5/S1 disc. According to Edwards,^92^ more than 60% of ASD patients with LIV at L5 could present with L5/S1 disc degeneration, global sagittal malalignment and clinical deterioration. Theoretically, a long lever arm of the fixed segments imposed more sheer force on the distal level, contributing to the degeneration of distal junctional segments.^83,88,93^

Failure to restore optimum sagittal alignment predisposes distal junctional problems. Ghasemi and colleagues^81^ found that greater correction of thoracic kyphosis and less change of lumbar lordosis (negative postoperative sagittal alignment) was correlated with DJK. They assumed that DJK might be a compensatory mechanism that counteracts the overcorrected thoracic kyphosis to maintain sagittal alignment. According to Kown and colleagues,^83^ surgical management of the distal fixed segments might also be associated with the occurrence of DJK. Their retrospective study showed that 12 out of 13 DJF patients had circumferential fusion with anterior interbody bone grafting or prosthetic cages inserted at the levels immediately cephalad to the failure site. The hypothesis was that the additional stiffness conferred by solid circumferential fusion could subject additional stress to the caudal fixation point. Another surgery-side factor related with DJK is interruption of the ligamentous complex in the distal junctional levels as is also the case in PJK.^88,93,94^

In addition, several patient factors were reported to be associated with DJK. Arlet^88^ noticed that obesity, old age, osteoporosis, and positive sagittal malalignment were risk factors for developing junctional problems. Kwon^83^ found that 84.6% (11/13) of the patients with distal junctional problems had osteopenia or osteoporosis, which could partially explain progressive DJK due to cage subsidence, and screw migration or pull-out in the distal region. Moreover, the fixed flexion deformity due to severe osteoarthritis of the hip joint might cause positive sagittal malalignment after corrective surgery, which could subject significant sheer force to the L5/S1 segment when the LIV was selected at L5, leading to DJK.^83,88^

DJK could be associated with compromised clinical outcome and HRQOL.^79,83,93^ According to Lowe,^79^ DJK can be a source of pain, imbalance, and poor cosmesis. Kwon^83^ speculated that for adult patients with DJF, low back pain was frequently encountered, and that a progressive sensation of “leaning forward” is characteristic of this clinical problem. To the best extent of our knowledge, no English literature has evaluated the quality-of-life measures for DJK patients. Biomechanically, loss of lordosis in the caudal junctional disc of a long-spanned instrumentation could cause significant adverse effects on the global sagittal alignment. It has been thoroughly reported that suboptimal sagittal spinopelvic alignment secondary to distal sagittal deformity is associated with poor quality-of-life measures.^95,96^ In order to maintain sagittal balance, the body adapts several compensation mechanisms including thoracic hypokyphosis (or even lordosis), pelvic retroversion, hip and knee flexion, as well as ankle dorsiflexion to counteract the regional kyphosis, which inevitably causes muscle fatigue and mechanical pain, and limits patients’ capability of independent daily life.^95-99^ Depending on the type of failures in the distal junctional segments, patients may complain of different levels of pain.^88^ Those with accompanying lumbar stenosis could present with sensory deficit of lower limbs or even radiculopathy.

#### Strategies for Prevention

So far, no evidence-based study to date has systematically described the strategies or options to avoid DJK in ASD patients. Based on previous literatures and our experiences, attention should be paid to the following aspects to reduce the incidence of DJK before and during corrective surgery for ASD:

(1) Try to restore harmonious sagittal spinopelvic alignment. Optimal age-adjusted and individualized postoperative sagittal alignment is of critical importance as it correlates with the incidences of DJK and other postoperative mechanical failures.^83,84,100^
(2) Meticulously selecting the distal fusion level. Recent years have witnessed the debate on the optimum selection of LIV to a trade-off strategy between preserving lumbar range of motion and minimize the incidence of distal junctional problems in patients with thoracic/thoracolumbar hyperkyphosis.^80,81,101-103^ Generally, for adult patients with thoracic/thoracolumbar hyperkyphotic deformity, appropriate selection of LIV should be based on an individualized and comprehensive evaluation of the length of kyphotic deformity, the desired sagittal alignment and the position of LIV in relation to the sagittal stable zone.^80,81,101^ For patients with lumbar spinal deformity, the reported occurrence of distal junctional problems in patients with distal fusion to L5 or S1 was higher than those with iliac fixation.^104-107^ Therefore, pelvic fixation via iliac or S2 alar-iliac screws has been generally recommended for spinal deformities involving the distal lumbar segments.^108,109^ Extending distal fixation to the pelvis may be recommended for patients with (a) more than 5 fusion segments; (b) three-column osteotomy in the lower lumbar segments; (c) lumbosacral deformity or pelvic obliquity; (d) spondylolisthesis or signs of instability in the lower lumbar segments; (e) weakness of muscles around spine and pelvis-hip; (f) osteoporosis; (g) significant biplanar (coronal & sagittal) imbalance.
(3) When distal fixation was planned at the pelvis, circumferential fusion with interbody cages is recommended in the distal segments to prevent DJF.^110^
(4) Preserve the posterior ligament complex of the distal fused segments.
(5) Rod contouring should be matched with the distal unfused curvature.^111^
(6) Postoperative care including use of brace for several months after surgery, instruction of patients’ habits (avoid lifting weights, adapt erect standing & sitting position, etc.) and physical exercise may help reducing the incidence or halt the progression of DJK.^88,93^

#### Controversy and Knowledge Gaps

The measurement of distal junctional angle and the definition of DJK varied between studies. Lowe^79^ first described DJK as an angle ≥10°between the superior endplate of LIV and the inferior endplate of the adjacent distal vertebra. Meanwhile, long fusion to the lower lumbar spine and pelvis was frequently observed in ASD surgeries. As is demonstrated in Figure 1, although the distal junctional angle (0° of L4-L5 kyphosis) did not meet the criteria of DJK definition, revision surgery is still needed to correct the progressive positive sagittal imbalance. Therefore, a modification of the classical definitions of DJK is warranted to comprehensively reflect the distal junctional problems. In addition, although DJK was reported in several studies regarding its correlation with clinical outcome of ASD surgery, its clinical manifestations, radiological features, and indications for revision surgery were not investigated in-depth.

#### Research Questions in Need of Answering

Considering the relatively low occurrence, high revision rate and lack of systematic investigations of DJK, a multicenter prospective study is warranted to reveal its natural history in ASD patients. Comprehensive analysis of the clinical symptoms, quality-of-life metrics, as well as the radiological measurements at pre-operation, post-operation and longitudinal follow-up could help:

(1) to determine the appropriate definition of DJK;
(2) to identify the patient-related and surgery-related risk factors of DJK;
(3) to clarify the indications for revision surgery and surgical options for DJK.

## Conclusions

Late complications in adult spinal deformity surgery are important to recognize and avoid. Durability of spine reconstructions is important to improve the cost-effectiveness of care for adult deformity. We reviewed common complications including infection, pseudarthrosis and junctional pathology, and we identified areas that are priorities for further investigation. Multicenter international prospective studies are valuable for generalizability of results of study. Identifying appropriate predictor and outcome variables are also important for identifying factors, modifiable and fixed, that may be important to make a clinically important difference in outcomes. Machine learning and acquisition of large datasets enable inclusion of variables that extend beyond radiomics and surgical metrics, and include patient specific factors including social factors, genomics, proteomics, and metabolomics. Future studies to reduce late complications are important to improve value and outcome in adult deformity surgery.

## Notes

1. Maruo K, Berven SH: Outcome and treatment of postoperative spine surgical site infections: predictors of treatment success and failure. J Orthop Sci. 2014 May;19(3):398-404.

2. Bouloussa H, Mirza M, Ansley B, Jilakara B, Yue JJ: Implant Surface Technologies to Prevent Surgical Site Infections in Spine Surgery. Int J Spine Surg. 2023 Dec 27;17(S3):S75-S85.
